# Attention-Based Multimodal Framework for Athlete-Performance Analysis and Rehabilitation Monitoring Using Vision and Wearable Sensors

**DOI:** 10.3390/bioengineering13070718

**Published:** 2026-06-23

**Authors:** Mohammed Alonazi, Iqra Aijaz Abro, Maha Abdelhaq, Raed Alsaqour, Ahmad Jalal, Hui Liu

**Affiliations:** 1 Department of Information Systems, College of Computer Engineering and Sciences, Prince Sattam bin Abdulaziz University, Al-Kharj 16273, Saudi Arabia; mn.alonazi@psau.edu.sa; 2 Faculty of Computer Science and AI, Air University, E-9, Islamabad 44000, Pakistan; iqraabro04@gmail.com; 3 Department of Information Technology, College of Computer and Information Sciences, Princess Nourah bint Abdulrahman University, P.O. Box 84428, Riyadh 11671, Saudi Arabia; maha.ukm@gmail.com; 4 Department of Information Technology, College of Computing and Informatics, Saudi Electronic University, Riyadh 93499, Saudi Arabia; raed.ftsm@gmail.com; 5 Department of Computer Science and Engineering, College of Informatics, Korea University, Seoul 02841, Republic of Korea; 6 Guodian Nanjing Automation Co., Ltd., Nanjing 210003, China; 7 Jiangsu Key Laboratory of Intelligent Medical Image Computing, School of Artificial Intelligence (School of Future Technology), Nanjing University of Information Science and Technology, Nanjing 210044, China; 8 Cognitive Systems Lab, University of Bremen, 28359 Bremen, Germany

**Keywords:** biosensing devices, artificial intelligence, multimodal fusion, rehabilitation monitoring, athlete performance analysis, attention mechanism

## Abstract

Background: Advances in monitoring systems featuring wearable sensors, computer vision, and artificial intelligence (AI) have been increasingly used in sports science and rehabilitation practices as a means of movement pattern analysis, injury prevention, and training optimization. These technologies are becoming essential components of athlete-performance analysis and rehabilitation-monitoring systems designed to support biomechanical assessment, athlete development, and movement-quality evaluation. Athlete-performance analysis and rehabilitation monitoring increasingly rely on intelligent multimodal sensing systems capable of continuously evaluating movement quality, biomechanical patterns, training execution, and recovery progress. Human activity recognition (HAR) serves as a key enabling technology for these applications by providing automated assessment of human movement using wearable and vision-based sensing modalities. Therefore, the purpose of this study was to develop and evaluate an attention-based multimodal framework that integrates wearable inertial sensing and RGB video analysis for robust athlete-performance assessment and rehabilitation monitoring through accurate recognition of human movement patterns. Methods: Athlete-performance analysis and rehabilitation monitoring combining inertial sensor data and RGB-based visual information was introduced. Inertial signals were segmented with adaptive windowing, whereas silhouette refinement was performed to analyze motion structures from visual inputs in support of athlete-performance analysis and rehabilitation monitoring. Temporal, spatial, and motion features such as trajectory, orientation, and skeleton-based space-time representations were calculated from multimodal inputs. The proposed framework was designed to capture complex movement dynamics associated with rehabilitation exercises and sports-related motion patterns across heterogeneous sensing environments. Extracted features were then combined and optimized with a multimodal feature fusion approach, while the Ranger optimization algorithm was utilized during the process. An attention-based deep learning classifier was implemented to classify movement activities. Results: The results showed that the proposed framework reached accuracy scores of 88.40% and 87.96% on the VIDIMU dataset and the UTD-MHAD dataset respectively. Recognition performance across both inertial and vision-based modalities provided greater robustness than single-modality solutions. The integration of wearable sensing and computer vision modalities further improved the ability of the framework to analyze complex movement behaviors under varying execution conditions and environmental variations. Conclusion: The proposed multimodal framework provides a foundation for intelligent athlete-performance and rehabilitation-monitoring systems by integrating wearable sensing, computer vision, and attention-based artificial intelligence for robust movement analysis. The findings highlight its potential to support biomechanical assessment, movement-quality evaluation, training-performance monitoring, rehabilitation tracking, and injury-risk management in modern sports and healthcare environments.

## 1. Introduction

Sports performance assessment and rehabilitation monitoring have increasingly adopted wearable sensing, computer vision, and artificial intelligence technologies to objectively evaluate movement quality, biomechanical efficiency, training execution, and recovery progression. The increasing integration of intelligent sensing technologies [1] into sports and rehabilitation environments has transformed the way movement behavior, biomechanical efficiency, and physical performance are evaluated in both clinical and athlete-centered settings. Reliable detection of movement patterns and rehabilitation-related exercises can provide valuable information on mobility, motor function recovery, biomechanical performance, and overall physical fitness [2]. Such information can also support higher-level assessments including movement-quality evaluation, rehabilitation progress monitoring, fatigue-related movement adaptation analysis, and injury-risk management in athlete populations. Slight differences in movement execution can serve as indicators of fatigue, impaired motor coordination, movement inefficiency, and increased injury risk. In sports science applications, accurate activity recognition also plays an important role in training optimization, workload assessment, and athlete development through data-driven performance evaluation. Furthermore, multimodal monitoring technologies are increasingly being adopted in athletic environments to quantify movement efficiency, evaluate biomechanical performance, detect technique deviations, monitor training loads, and support injury-prevention strategies. Therefore, robust activity recognition frameworks constitute an important component of modern athlete-performance analysis systems. Thus, the introduction of robust athlete-performance analysis and rehabilitation-monitoring systems allows clinicians, rehabilitation specialists, sports scientists, and performance analysts to make evidence-based decisions and monitor movement behavior over extended periods.

Despite the remarkable achievements in the domain, obtaining satisfactory recognition results in complex and unconstrained environments remains a challenge. Variations in movement patterns between individuals, noise or incompleteness in sensor-based data, and occlusions, lighting conditions, and viewpoint variations in vision-based sensing systems introduce significant challenges in recognizing and classifying movement patterns. Such limitations become particularly critical in sports and rehabilitation scenarios, where movement execution may vary considerably depending on intensity, fatigue, recovery stage, or environmental conditions. Hence, unimodal sensing approaches are often insufficient when dealing with complex movement analysis tasks, as a single sensing modality may fail to capture the complete spatio-temporal characteristics of human motion.

Multimodal sensing approaches have therefore attracted considerable attention in recent years. The combination of inertial sensing with vision-based modalities enables complementary representation of human movement, where inertial sensors capture temporal motion dynamics while RGB-based visual information represents spatial features and body motion. The integration of wearable sensing and computer vision technologies has shown significant potential for sports-performance assessment, rehabilitation evaluation, biomechanical analysis, and movement-quality assessment [3]. However, several challenges remain unresolved, including multimodal feature integration, extraction of discriminative spatio-temporal representations, and robust generalization across different datasets and movement conditions [4]. In modern sports science, athlete-performance assessment extends beyond traditional performance metrics and increasingly incorporates continuous monitoring of movement patterns, workload, biomechanical efficiency, and rehabilitation status. Multimodal HAR frameworks provide a practical mechanism for capturing these movement-related indicators through integrated wearable and vision-based sensing technologies.

At the same time, recent developments in artificial intelligence and deep learning have significantly expanded the capabilities of athlete-performance analysis and rehabilitation-monitoring systems. Deep learning models incorporating attention mechanisms enable effective modeling of complex spatio-temporal dependencies in sequential multimodal data and provide improved representation learning and recognition performance [5]. In addition, modern optimization algorithms improve the training process of deep learning models by accelerating convergence, enhancing feature separability, and increasing the robustness of learned representations [6]. These advances have accelerated the adoption of intelligent activity recognition frameworks capable of supporting data-driven decision-making in sports performance analysis, rehabilitation engineering, and digital healthcare platforms [7].

Despite substantial progress in multimodal human activity recognition, several challenges remain. Existing approaches often emphasize sensor fusion and classification performance while providing limited consideration of modality-specific preprocessing, adaptive activity segmentation, and the integration of complementary motion representations. In addition, many studies rely predominantly on deep-learned features, potentially overlooking informative biomechanical characteristics related to movement complexity, energy variations, and motion regularity that may be particularly relevant for athlete monitoring and rehabilitation assessment. Furthermore, effectively combining heterogeneous visual and inertial sensing modalities remains challenging because of differences in temporal dynamics, motion representations, and noise characteristics. To address these challenges, this study proposes an attention-based multimodal framework that integrates wearable inertial sensing and RGB video analysis to support athlete-performance analysis and rehabilitation monitoring through robust recognition of biomechanical and spatio-temporal movement patterns.

The proposed framework incorporates modality-specific preprocessing techniques, adaptive segmentation of inertial data, silhouette refinement for RGB inputs, multimodal feature extraction, feature fusion, and optimization using the Ranger optimizer. An attention-based deep learning architecture is employed to model spatio-temporal dependencies in multimodal data and perform activity classification. By integrating multimodal sensing and attention-guided learning, the framework aims to improve the robustness and reliability of athlete-performance analysis and rehabilitation monitoring. The performance of the framework is validated through extensive experimental evaluation on benchmark multimodal datasets containing rehabilitation-related and biomechanical movement activities. Unlike many existing multimodal rehabilitation systems that primarily focus on sensor fusion and classification performance, the proposed framework integrates modality-specific preprocessing, adaptive activity segmentation, hybrid handcrafted and deep feature extraction, multimodal feature fusion, Ranger-based optimization, and attention-guided learning within a unified architecture. This comprehensive design enables the framework to exploit complementary biomechanical and spatio-temporal information from wearable inertial sensors and RGB video data, thereby providing a more robust solution for athlete-performance analysis and rehabilitation monitoring.

The major contributions of this study are summarized as follows:

- We propose a unified multimodal HAR framework that integrates inertial sensing and RGB-based visual information through modality-specific preprocessing, hybrid feature extraction, multimodal fusion, and attention-guided classification within a single end-to-end architecture.
- We introduce a hybrid representation strategy that combines deep inertial features learned by 1D-CNN with motion-complexity descriptors including Teager–Kaiser Energy Operator, Fractal Dimension, and Permutation Entropy, while simultaneously integrating silhouette-based and skeleton-based visual representations.
- We employ adaptive activity-aware segmentation and silhouette-refined pose modeling to improve the quality and consistency of multimodal representations extracted from heterogeneous sensor streams.
- We incorporate Ranger optimization within the multimodal learning pipeline to enhance convergence stability and feature discrimination during attention-based classification.
- We validate the proposed framework on two benchmark multimodal datasets (VIDIMU and UTD-MHAD), demonstrating robust recognition performance and applicability to athlete-performance analysis, training-monitoring applications, rehabilitation assessment, movement-quality evaluation, and injury-risk monitoring scenarios.

### Research Objective and Hypotheses

The primary objective of this study is to develop, implement, and experimentally validate a unified multimodal framework for athlete-performance analysis and rehabilitation monitoring using wearable inertial sensing and RGB video analysis that integrates wearable inertial sensing and RGB video analysis for athlete-performance assessment and rehabilitation monitoring. The framework is designed to improve activity-recognition accuracy, robustness, and generalization by combining modality-specific preprocessing, hybrid handcrafted and deep feature extraction, multimodal feature fusion, Ranger-based optimization, and attention-guided classification.

To guide the development and evaluation of the proposed framework, the following research hypotheses are formulated:

**H1.**  *The integration of RGB visual information and inertial sensor data provides higher activity-recognition performance than conventional single-modality learning approaches because of the complementary spatial and temporal information captured by the two sensing modalities.*

**H2.**  *The integration of handcrafted biomechanical descriptors (Teager–Kaiser Energy Operator, Fractal Dimension, and Permutation Entropy) with deep learned representations is expected to provide complementary motion information that enhances multimodal feature representation and activity-recognition performance.*

**H3.**  *Attention-guided multimodal learning combined with Ranger optimization improves classification robustness and feature discrimination, resulting in enhanced recognition performance across benchmark multimodal activity-recognition datasets.*

## 2. Literature Review

### 2.1. Health Monitoring and Rehabilitation Using Single Sensors

Systems that employ a single modality such as IMUs, Electrocardiogram (ECG), Electromyography (EMG), or vision-based approaches for healthcare and rehabilitation purposes have become increasingly popular in terms of implementation and application. IMU-based wearable sensors in rehabilitation have been studied intensively lately and proved to be successful. Mundt et al. [8] employed IMUs to conduct a study concerning lower limb rehabilitation and reported high correlations with traditional gait assessment systems. In terms of cardiovascular monitoring, ECG-based systems prevail among single-sensor systems. Moreover, Hannun et al. [9] proposed an approach to ECG monitoring with deep learning technology to detect arrhythmia with cardiologists’ accuracy. In addition, muscle monitoring based on EMG systems is common in medical practice. In this way, Yang et al. [10] proposed using EMG systems to monitor muscle activity and provide biofeedback after stroke to facilitate patients’ recovery. Nevertheless, one of the problems with EMG monitoring is the presence of noises and the fact that the exact location of the electrodes plays a critical role in obtaining correct results. Similarly, Kiper [11] described EMG-based neurorehabilitation systems, emphasizing the lack of robustness across subjects. On the other hand, vision-based single-sensor systems have developed tremendously. Chen et al. [12] introduced the approach of remote physiotherapy using single monocular RGB cameras to track patients’ motion using pose estimation algorithms. However, vision-based systems depend heavily on light conditions and occlusions.

However, despite numerous advantages, such approaches are limited by poor context awareness, robustness, and inability to consider complex physiological processes.

### 2.2. Health Monitoring and Rehabilitation Using Multimodal Sensors

Multimodal sensor systems leverage data collected from different kinds of sensors, e.g., IMUs, cameras, EMG, ECG, and pressure sensors, to gain a more complete picture of patients’ health and rehabilitation status. This technique can help to overcome many disadvantages of single-modal solutions by enhancing the accuracy, reliability, and interpretability of the obtained data. Many recent publications focus on the effectiveness of multimodal approaches in monitoring patients’ rehabilitation activities. Thus, for example, Ordóñez & Roggen [13] employed a deep convolutional and LSTM recurrent architecture (DeepConvLSTM) for human activity recognition. Although their study relied on a single sensing modality, namely multiple inertial measurement units distributed across different body locations (the OPPORTUNITY and Skoda datasets), rather than heterogeneous modalities such as inertial and visual data, their work demonstrated that deep learning can effectively fuse heterogeneous sensor streams and thereby provided a foundational basis for subsequent multimodal recognition systems combining inertial sensors and video. Multimodal healthcare and rehabilitation systems based on various types of bio-signals and physiological parameters become a preferable option compared to unimodal solutions due to their high efficiency in decision-making support. According to studies, the combination of various sensors, e.g., IMU, EMG, and ECG devices, ensures much higher results in the monitoring of rehabilitation and recovery. In particular, Szabo et al. [14] indicate that this type of system transformed from purely monitoring solutions to intelligent therapeutic tools providing continuous feedback and adaptive assistance in patients’ rehabilitation, particularly in cases related to musculoskeletal and neurological conditions. Moreover, Munteanu et al. [15] demonstrate that combining bio-signals with the motion of body parts provides an opportunity to understand the progress of rehabilitation more comprehensively. The combination of EMG, ECG, and inertial data makes it possible to monitor both neuromuscular and cardiovascular changes continuously and adjust rehabilitation programs to these changes. The synchronization of sensors helps align the internal physiological parameters with the external activity of the human body to achieve better rehabilitation results. Furthermore, Gu et al. [16] found that in predicting various outcomes, e.g., the risk of falls and mobility decline, multimodal wearable systems exhibit higher efficiency than single-sensor solutions. The main reason behind this result is that multimodal wearable systems allow collecting and analyzing additional information from various domains. At the same time, the authors note that multimodality significantly expands the role of machine learning in health monitoring and predicting. Finally, Gaikwad et al. [17] develop an application-oriented approach to sensor systems for health monitoring using the fusion of data obtained with wearable sensors and EEG. They developed a highly efficient system with predictive properties and advanced signal processing for the purpose of health feedback. Kaur and Verma [18] designed an emergency detection system that integrates data from several biosensors including ECG, heart rate, respiration rate, and blood alcohol concentration sensors. The focus of their work was the design of a highly performant algorithm for real-time processing of multimodal data.

Despite the potential of multimodal sensing in healthcare monitoring, rehabilitation, and HAR applications as presented in the above-reviewed literature, some limitations are yet to be addressed. In general, current techniques focus more on sensor data fusion and classification processes rather than integrating modality-specific processing techniques, segmenting activities from raw sensor readings, optimally extracting activity-specific features, and attention-based learning of multimodal signals into a single framework. Moreover, current multimodal HAR techniques heavily depend on deep learning approaches, ignoring the significance of complementary handcrafted motion features that capture the biomechanics of motions, energy changes, and abnormalities associated with rehabilitation tasks and athletes’ monitoring applications. In addition, fusing heterogeneous modalities such as visual information and inertial sensors is complicated owing to varying temporal properties, noise distributions, and different motion representations. Inspired by these limitations, an attention-based multimodal framework is proposed to support athlete-performance analysis and rehabilitation monitoring through robust recognition of movement patterns, biomechanical behaviors, and activity execution using wearable and vision-based sensing modalities.

## 3. Materials and Methods

**Figure 1:**
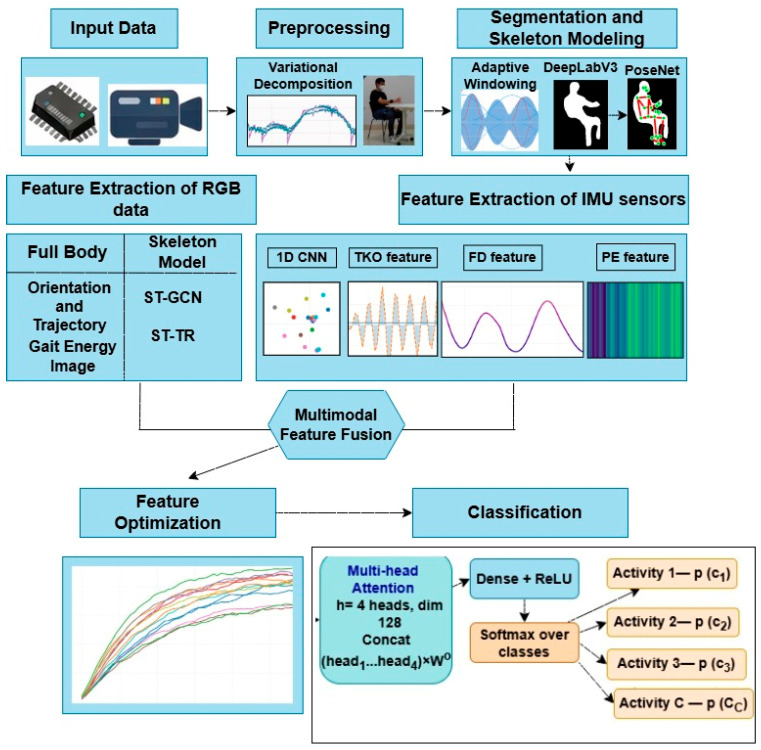
The system architecture of the proposed model.

The suggested framework uses visual and inertial data for athlete-performance assessment, rehabilitation monitoring, and movement pattern analysis, where visual data comes from RGB streams, and the system uses two databases, VIDIMU and UTD-MHAD, to test the algorithm’s effectiveness and generalizability. For the former, the RGB stream gets silhouettes generated and refined to eliminate background noise, whereas the latter involves cleaning and segmenting using adaptive windows to ensure temporal consistency. Then, discriminative features are obtained from each dataset, which includes spatial-movement information, such as orientations, trajectories, and skeletons from the visual dataset and temporal information from the inertial data that represent movement intensity and sequence dynamics. These features get fused using the multimodal fusion technique to exploit the synergies between different types of features and then optimized using the Ranger optimizer for effective convergence and feature separability. Finally, the fused multimodal features are processed by an attention-based classification network consisting of a multihead self-attention module followed by fully connected classification layers. In addition, 1D-CNN is employed for inertial feature extraction, while ST-GCN and spatial–temporal transformer (ST-TR) models are used to learn skeleton-based visual representations. The overall architecture of the system is shown in Figure 1.

The suggested architecture attempts to solve several challenges faced by multimodal movement-analysis and rehabilitation-monitoring systems, which include sensor noise, activity-duration variability, heterogeneous feature representation, multimodal redundancy, and spatio-temporal dependency. Each component of the framework serves a specific role in the processing workflow. The purpose of preprocessing is data enhancement, while the hybrid feature extraction step ensures that complementary motion properties of two different modalities are exploited. Multimodal fusion makes use of cross-modal information, Ranger optimization contributes to convergence stability, and the attention-based classification step focuses on discriminative feature selection.

The proposed framework consists of modality-specific preprocessing, inertial and visual feature-extraction modules, multimodal feature fusion, Ranger-based optimization, and an attention-guided classification network. For inertial sensing, a three-stage 1D-CNN employing 64, 128, and 256 convolutional filters is used to learn hierarchical temporal representations. Visual information is represented through silhouette-based and skeleton-based processing streams utilizing DeepLabV3, ST-GCN, and ST-TR models. The resulting multimodal feature representation is projected into a 512-dimensional fused feature space and subsequently processed by a multihead self-attention module with four attention heads and an embedding dimension of 128 before final classification. The training configuration and hyperparameter settings are summarized in Table 1.

### 3.1. Sensor Filtration

In this phase, inertial sensor outputs are subjected to preprocessing using variational mode decomposition (VMD). VMD was selected because inertial measurements frequently contain motion artifacts and high-frequency noise that may degrade feature quality. Compared with conventional filtering approaches, VMD enables adaptive decomposition of the signal into frequency-specific modes while preserving motion-related information that is important for activity recognition. Contrary to linear filter designs, VMD breaks down the original signal into IMFs with finite bandwidth according to a specified number of IMFs and exploits the adaptability of separating signals depending on the spectrum distribution. For an input signal δ(t) s(t) from an inertial sensor, VMD finds an estimate for each IMF $u_{k}$ and the center frequency $\omega_{k}$ by solving an optimization problem in which the sum of bandwidths of IMFs is minimized:

(1) $$\underset{\left\{u_{k}\},\{\omega_{k}\right\}}{min}\sum_{k}{∥\partial_{t}\left[\left(\delta \left(t\right)+\frac{j}{\pi t}\right)*u_{k}\left(t\right)\right]e^{-j\omega_{k}t}∥}_{2}^{2}$$

subject to $\sum_{k}u_{k}\left(t\right)=s\left(t\right)$, where each mode represents a specific frequency sub-band of the original signal. In the proposed implementation, inertial signals from each axis and joint channel are independently decomposed using K = 3 modes with a penalty parameter α = 2000, ensuring effective separation of high-frequency noise from meaningful motion components. The reconstructed signal is obtained by aggregating the dominant modes while discarding residual high-frequency components associated with noise.

**Figure 2:**
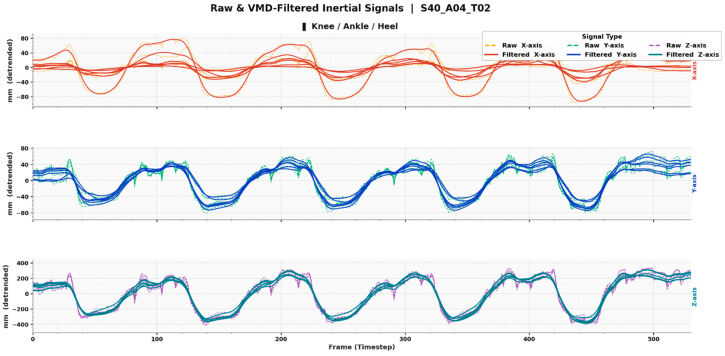
The raw vs. VMD-filtered inertial signals along the X, Y, and Z axes, respectively, for the Knee–Ankle–Heel joint group. The dashed curves represent the raw signals, while the solid curves illustrate the filtered outputs, demonstrating effective noise suppression and preservation of motion patterns over VIDIMU dataset.

The filter design methodology is adopted for all inertial signal sources, that is, several body joints and their corresponding X, Y, and Z axis values. Furthermore, before applying other operations on the obtained data, de-trending is performed to remove any DC offset and stabilize time domain variations. The efficacy of the proposed VMD filtering method is validated using comparative visualization of the original and filtered waveforms, where noise components are effectively eliminated while retaining important transition components and time-domain continuity as illustrated in Figure 2.

### 3.2. Adaptive Signal Windowing

To effectively segment the inertial signal into relevant activities, adaptive windows are used rather than fixed windows. Contrary to the standard technique that uses predetermined window sizes, the technique presented dynamically segments data according to some rules based on signal attributes and activity transitions. More precisely, windowing begins at the start point and continues until one of three criteria is met: (i) an activity class label change, denoting a shift in motion, (ii) missing or invalid sensor readings, or (iii) reaching a maximum window size. This way, it can be ensured that every window will contain a homogenous sequence of activity. While ensuring that windowing takes place in a timely manner without producing overly small windows, a minimum window size requirement is also set, and the maximum is restricted to 150 samples. Formally, window $W_{i}$ is defined as

(2) $$W_{i}=\left\{x_{t}|t=t_{s},\ldots ,t_{e}\right\},\;\mathrm{subject}\;\mathrm{to}\;L_{min}\leq (t_{e}-t_{s})\leq L_{max}$$

where $t_{s}$ and $t_{e}$ denote the start and end indices of the window, and $L_{min}$ and $L_{max}$ represent the minimum and maximum window lengths, respectively.

**Figure 3:**
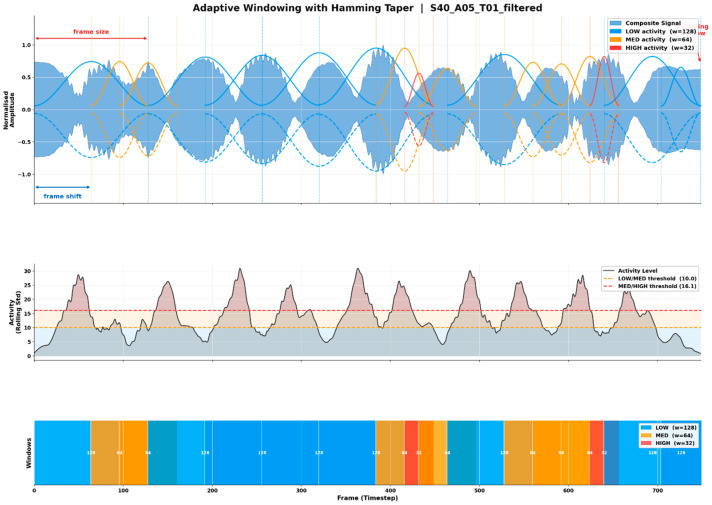
Adaptive windowing with Hamming taper showing composite inertial signal, activity intensity levels with thresholds, and resulting variable-length windows categorized into low, medium, and high activity over VIDIMU dataset.

This adaptive segmentation preserves the temporal structure of activities while preventing fragmentation or over-smoothing of motion patterns. Additionally, it ensures robustness against irregularities in sensor data and variations in activity duration. Each segmented window is subsequently stored as an independent sample for downstream feature extraction and classification. Visualization of the segmented windows as shown in Figure 3 demonstrates that the adaptive approach effectively aligns with activity boundaries while maintaining continuity in signal magnitude, thereby improving the quality of temporal representation for human activity recognition tasks.

### 3.3. Silhouette Detection and Skeleton Modeling

Silhouette extraction is performed using a deep learning-based semantic segmentation approach to obtain accurate human foreground masks from RGB frames. Specifically, a pretrained DeepLabV3 model with a ResNet-101 backbone is employed to perform pixel-wise classification, where each pixel is assigned a probability of belonging to the human class. The input RGB frames are first normalized using ImageNet statistics and resized to a fixed resolution to ensure computational efficiency and consistency during inference. The segmentation network produces a probability map *P(x,y)* for each pixel, and the human silhouette is obtained by applying a confidence-based thresholding operation:

(3) $$S\left(x,y\right)=\;\left\{\begin{array}{c} 255,\\ 0, \end{array}\right.\begin{array}{c} \;\;\;\;if\;P(x,y)\geq \tau \\ \;\;\;otherwise \end{array}$$

where $S\left(x,y\right)$ denotes the binary silhouette mask, P(x,y) is the predicted probability for the human class, and τ is the confidence threshold (set to 0.6).

To further refine the extracted silhouettes, morphological operations are applied, including closing small holes within the foreground region and opening to remove spurious noise and thin artifacts. Additionally, connected component analysis is performed to eliminate small, isolated regions below a predefined area threshold, ensuring that only the primary human body region is retained. This post-processing step significantly improves the structural integrity and continuity of the silhouette.

Skeletonization is performed to extract structured human pose representations from silhouette-enhanced RGB frames using the MediaPipe Pose framework. This model detects a set of anatomically significant body landmarks, including key joints such as the shoulders, elbows, wrists, hips, knees, and ankles, forming a 2D skeletal graph that captures human posture and motion dynamics. Given an input frame, the model predicts normalized landmark coordinates $\left(x_{i},y_{i},z_{i}\right)$ along with a visibility confidence score for each joint. To improve localization accuracy, the region of interest is first constrained using the silhouette mask, and the detected landmarks are remapped to the original image coordinates after scaling and bounding box transformation. In addition to directly detected keypoints, auxiliary joints are computed to enhance structural representation. For example, the neck position is estimated as the midpoint between the left and right shoulder landmarks:

(4) $$\left(x_{neck},y_{neck}\right)=\left(\frac{x_{LS}+x_{RS}}{2},\frac{y_{LS}+y_{RS}}{2}\right)$$

where $\left(x_{LS},y_{LS}\right)$ and $\left(x_{RS},y_{RS}\right)$ correspond to the left and right shoulder coordinates, respectively. Similarly, central body alignment can be approximated using midpoints between bilateral joints such as hips or eyes to improve pose stability.

**Figure 4:**
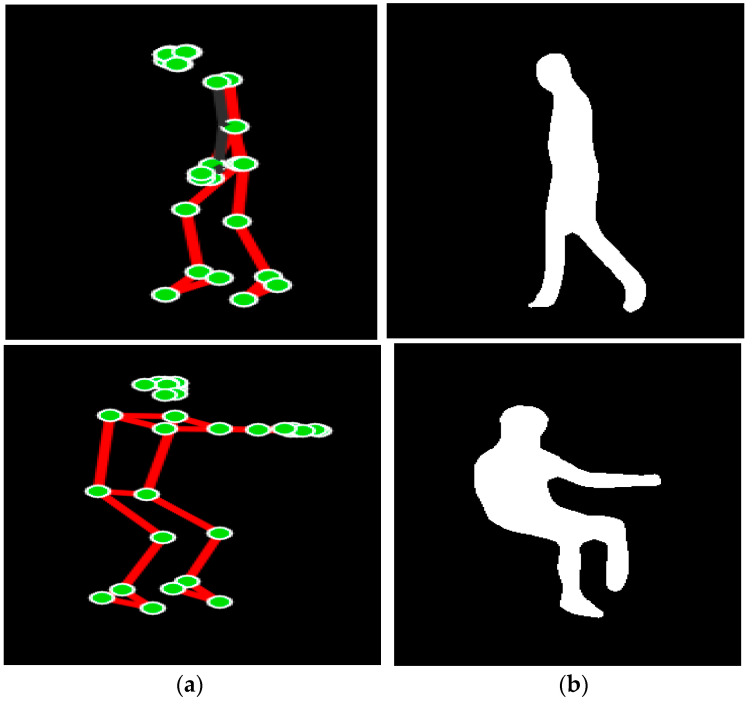
Results of skeleton modeling and silhouette extraction with (**a**) skeleton model, (**b**) silhouette extraction over VIDIMU dataset.

The final skeletal model is constructed by connecting predefined landmark pairs to form a kinematic chain representing the human body. These connections encode spatial relationships between joints, enabling effective modeling of motion trajectories and joint dependencies. Low-confidence landmarks are filtered or assigned reduced importance to ensure robustness against occlusions and detection noise. The resulting skeleton representation provides a compact and discriminative abstraction of human motion, facilitating efficient spatio-temporal feature extraction and improving the performance of downstream activity recognition tasks. Figure 4 illustrates the output of this process, showing the extracted skeleton model (a) and the corresponding refined silhouette (b) over the VIDIMU dataset.

### 3.4. Feature Extraction for Inertial-Based Sensor

In the feature extraction phase, discriminative representations were obtained from IMU data to represent the dynamics of motions associated with daily behaviors and falls. Four types of features were used in this study to represent temporal, spectral, and nonlinear properties, including 1D-CNN, Teager–Kaiser Energy Operator (TKEO), Fractal Dimension (FD), and Permutation Entropy (PE). The 1D-CNN can learn local temporal patterns from the raw data sequence, while TKEO is able to emphasize abrupt changes in energy. FD features can be utilized to quantify the complexity and self-similarity of inertial signals, and the PE represents signal complexity.

A combination of handcrafted and deep features was employed because these representations capture complementary aspects of human motion. Deep features learned by 1D-CNN effectively model hierarchical temporal patterns, whereas handcrafted descriptors such as TKEO, Fractal Dimension, and Permutation Entropy provide explicit information regarding energy fluctuations, movement complexity, and signal irregularity that may not be fully captured by learned representations alone.

#### 3.4.1. The 1D-CNN

**Figure 5:**
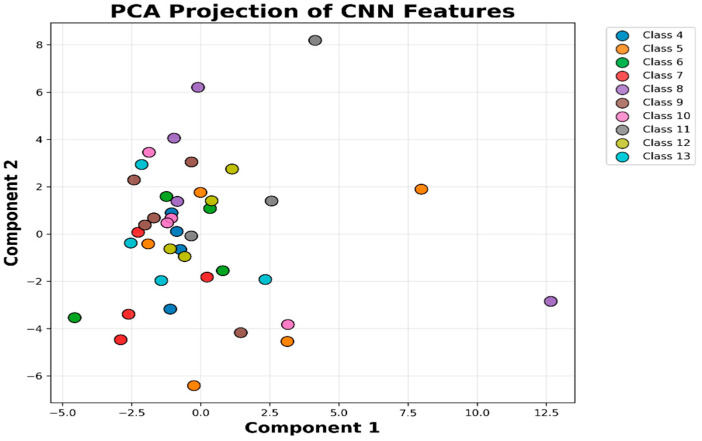
PCA projection of 1D-CNN-extracted deep features onto two principal components, illustrating the distributional spread of 10 activity classes (Classes 4–13) from the VIDIMU dataset.

The one-dimensional Convolutional Neural Network (1D-CNN) is used to learn hierarchical temporal features from inertial sensor signals, thereby addressing the limitation of handcrafted features in representing complex motion dynamics. In contrast to traditional methods, the 1D-CNN automatically extracts discriminative patterns directly from the raw multivariate time-series data, providing robust representations for smooth as well as sharp motions as illustrated in Figure 5.

In the first place, the IMU data are split into overlapping windows $X\in R^{T\times F}$, where T and F correspond to temporal length and sensor channels, respectively. This data is fed to a number of convolutional blocks comprising Conv1D, Batch Normalization (BN), and MaxPooling layers. The increasing number of filters (64, 128, 256) combined with stacked convolutional and pooling operations enables the network to learn hierarchical temporal representations at progressively larger receptive fields. Batch Normalization (BN) improves training stability, while MaxPooling reduces temporal dimensionality and increases the effective temporal context captured by deeper layers. BN improves the stability of training while MaxPooling further decreases the temporal dimensions of feature maps. GAP finally aggregates the feature maps in order to get the representation that is invariant to temporal changes, which is further mapped using dense projections to a fixed-dimensional space. The extracted deep feature vector $z\;\in R^{128}$ is computed as

(5) $$z=\sigma \left(W\cdot \frac{1}{T^{\prime }}\sum_{t=1}^{T^{\prime }}\phi_{3}(\phi_{2}(\phi_{1}\left(X\right)){)}_{t}+b\right)$$

where $\phi_{k}(\cdot )=\mathrm{BN}({\mathrm{Conv}}_{k}(\cdot ))$ denotes the k-th convolutional transformation, $T^{\prime }$ is the reduced temporal dimension after pooling, and σ(⋅) is the ReLU activation. W and b are learnable parameters of the dense layer. Each window is processed independently in inference mode to generate a 128-dimensional feature vector, preserving its label for downstream classification. This approach captures multi-scale temporal abstractions and enhances separability between normal and fall activities, while maintaining computational efficiency for large-scale inertial datasets.

It should be noted that the 1D-CNN is employed as a local temporal feature extractor rather than as a standalone sequence-recognition architecture. The objective of this module is to learn discriminative short-term motion characteristics from inertial signals, while longer-duration activity information is preserved through adaptive segmentation and further represented through handcrafted temporal descriptors, multimodal feature fusion, and the attention-based classification module. Therefore, recognition performance depends on the combined multimodal representation rather than solely on the temporal receptive field of the convolutional kernels.

#### 3.4.2. Teager–Kaiser Energy Operator (TKEO)

The Teager–Kaiser Energy Operator (TKEO) is utilized to obtain instantaneous energy fluctuation from inertial signals by overcoming the shortcomings of existing energy features, which do not account for coupled amplitude and frequency fluctuations. TKEO provides a nonlinear estimate of the instantaneous energy associated with local amplitude and frequency variations in the signal, making it highly sensitive to transient events and motion changes.

**Figure 6:**
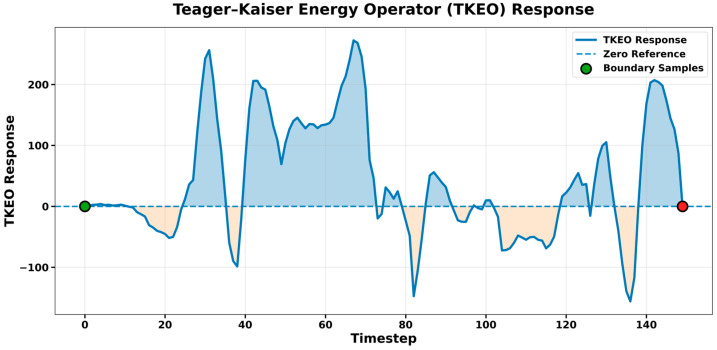
TKEO response for a representative IMU channel (pelvis_x). Positive and negative responses reflect local amplitude–frequency interactions, while boundary samples are zero-padded during computation.

Firstly, the multivariate inertial signal is segmented into frames $X\;\in \;R^{T\times F}$, where T stands for time steps, and F refers to sensor channels. For each channel, TKEO computation is done independently and produces an enhanced energy signal that highlights the temporal aspects of the signal and the fast variations in the signal. This emphasizes transient energy variations and rapid changes in motion dynamics that characterize dynamic human movements. The TKEO-derived feature for each channel is defined as

(6) $$E_{f}\left[n\right]=x_{f}^{2}\left[n\right]-x_{f}\left[n-1\right]\cdot x_{f}\left[n+1\right]$$

where $x_{f}[n]$ denotes the signal value of the *f*-th channel at time index *n*. Boundary values are padded with zeros to preserve dimensional consistency. It should be noted that the TKEO output is not constrained to be strictly non-negative for arbitrary discrete signals. Although commonly interpreted as an estimate of instantaneous energy, the discrete TKEO is not mathematically constrained to be non-negative and may therefore produce negative responses in regions characterized by rapid oscillations, noise, or local signal irregularities. In the present implementation, boundary samples are handled through zero-padding, such that unavailable neighboring samples at the beginning and end of each segment are replaced with zeros to preserve dimensional consistency. In matrix form, the transformed feature representation is expressed as

(7) $$E=\Psi \left(X\right)=X⊙X-X_{\left(-1\right)}⊙X_{\left(+1\right)}$$

where ⊙ denotes element-wise multiplication, $X_{-1}$ and $X_{+1}$ represent backward and forward shifted versions of X, respectively. Each window is processed independently to generate TKEO feature representations that capture instantaneous energy dynamics and local temporal variations. The TKEO response follows changes in the underlying inertial signal because it is computed from local interactions between neighboring samples. Consequently, abrupt changes in motion dynamics, acceleration patterns, or signal curvature generate pronounced positive or negative responses. These characteristics emphasize impulsive movement behavior and improve discrimination between normal activities and fall-related events, as illustrated in Figure 6.

#### 3.4.3. Fractal Dimension (FD)

The Fractal Dimension (FD) is used to characterize inertial signal complexity and self-similarity in terms of a reliable metric of nonlinear dynamics, which can hardly be evaluated using linear statistical measures. FD is a geometrical measure of the underlying pattern of the signal and proves particularly useful in detecting chaos in fall motions and excluding regular motions. At the first step, the VMD-filtered and detrended inertial data matrix X obtained from the preprocessing stage (Section 3.1) is divided into windows. Consequently, the Fractal Dimension is computed from signals in which DC offsets and high-frequency noise components have already been substantially reduced. One channel of motion signal is defined as an average signal across all sensors, and then the FD is estimated via the Higuchi algorithm in the time domain. The Higuchi approach involves estimation of the signal length at several temporal scales, thereby considering the properties of motions that are invariant to scaling transformations. The use of preprocessed inertial signals is particularly important for FD estimation because excessive sensor noise and baseline drift may artificially alter signal complexity and lead to biased Fractal Dimension measurements. To consider the temporal variation, the sliding window technique is used, providing a temporal series of FD. The FD of segment is estimated according to the following formula.

(8) $$D=\frac{\partial logL\left(k\right)}{\partial log\left(1/k\right)},\;L\left(k\right)=\frac{1}{k}\sum_{m=0}^{k-1}\left(\frac{N-1}{\lfloor \left(N-m\right)/k\rfloor k}\sum_{i=1}^{\left\lfloor \left(N-m\right)/k\right\rfloor }\left|x\left[m+ik\right]-x\left[m+\left(i-1\right)k\right]\right|\right)$$

where L(k) denotes the average curve length at scale k, and N is the segment length. The FD is obtained as the slope of the log-log relationship between L(k) and 1/k.

**Figure 7:**
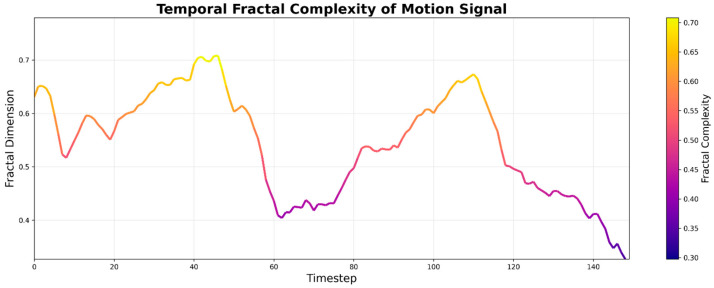
Temporal variation in Fractal Dimension representing motion complexity over time. Higher values indicate irregular, chaotic movements, while lower values reflect smoother and stable activity patterns over VIDIMU dataset.

Each time step is thus associated with a local Fractal Dimension value, forming a feature sequence $d\in R^{T}$ that captures temporal variations in motion complexity. Higher FD values indicate irregular, chaotic movements, while lower values correspond to smoother, periodic activities. This representation as shown in Figure 7 enhances sensitivity to abrupt transitions and nonlinear dynamics, improving discrimination between normal activities and fall events while maintaining computational efficiency.

#### 3.4.4. Permutation Entropy (PE)

**Figure 8:**
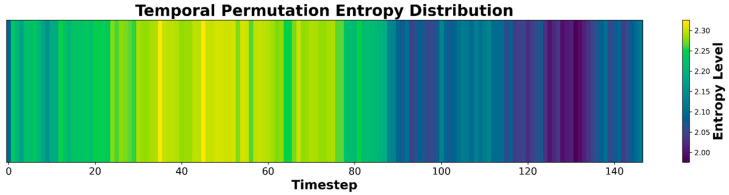
Temporal distribution of Permutation Entropy across the signal window. Higher entropy regions indicate increased motion complexity, while lower values reflect more regular patterns over VIDIMU dataset.

The Permutation Entropy (PE) is used to assess the dynamical complexities and irregularities of the inertial signal using ordinal patterns in the time series. In contrast to other measures of entropy, the Permutation Entropy technique does not rely on any specific signal properties and is invariant to nonlinear functions. Firstly, the inertial signal is divided into segments denoted by $X\in R^{T\times F}$. The univariate representative signal can be extracted from the set of sensors by calculating the average value. Then, for each segment, the embedding vector with dimension m and delay τ is built. The ordinal pattern for each embedding vector is evaluated according to their relative amplitudes. Permutation Entropy can be defined as

(9) $$H=-\sum_{i=1}^{m!}p_{i}{log}_{2}\left(p_{i}\right),\;p_{i}=\frac{\mathrm{count}\left(\pi_{i}\right)}{\sum_{j}\mathrm{count}\left(\pi_{j}\right)}$$

where $p_{i}$ represents the probability of the i-th permutation pattern $\pi_{i}$, and m! is the total number of possible ordinal patterns. Higher values of PE show greater randomness and erratic behavior of motion, whereas lower values are associated with more orderly motions (see Figure 8). Such a method is efficient at describing shifts from regular actions to unpredictable events like falling down, which increases the efficiency of discrimination without sacrificing computational speed.

### 3.5. Feature Extraction for Vision-Based Sensor

The process of feature extraction involves generating visual features from the RGB dataset using full-body and skeletal features to capture complementary motion characteristics. Full-body features can be generated using silhouettes based on DeepLabV3 and then using morphological filtering where the orientation, trajectory, and GEIs are calculated for the posture and motion features. On the other hand, skeletal features can be generated using PoseNet and modeled using spatio-temporal graph convolutional networks (ST-GCN) and spatial–temporal transformers (ST-TR), representing joint correlations and long-term temporal relationships. While ST-GCN models the local relationships between joints, the role of ST-TR is to model motion globally with attention mechanism.

Both silhouette-based and skeleton-based representations were utilized to capture complementary visual information. Silhouette descriptors provide global posture and motion characteristics, whereas skeleton-based representations encode joint-level kinematic relationships and temporal motion dynamics. Their combination enables a more comprehensive description of human movement patterns.

#### 3.5.1. Orientation and Trajectory Feature

**Figure 9:**
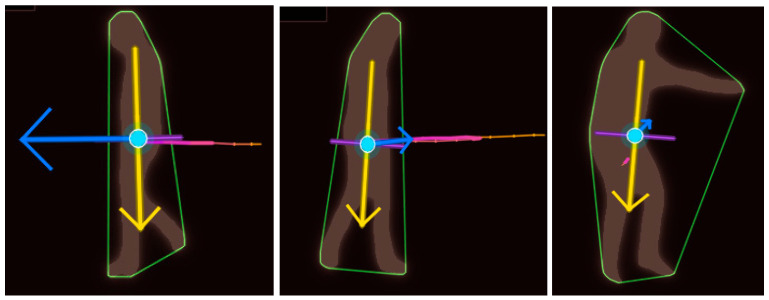
Body orientation (yellow axis) is estimated via PCA and centroid-based trajectory (blue vector) across frames. Motion direction and posture changes are captured jointly, highlighting spatial alignment and temporal displacement patterns.

Features for orientation and trajectories are derived from the binary images of silhouettes that represent the whole body (Figure 9). The body orientation is derived by employing the method of PCA on the set of pixel coordinates within the foreground region, where the primary direction of the body is defined by the major eigenvector. Orientation feature θ is given by

(10) $$\theta =atan2\left(v_{y},v_{x}\right)$$

where $\left(v_{y},v_{x}\right)$ denotes the dominant eigenvector obtained from PCA of the silhouette coordinates, and atan2 $\left(v_{y},v_{x}\right)$ is the two-argument arctangent function that determines the body orientation while preserving quadrant information. In addition, the trajectory of the subject is determined through the time variation in the silhouette center $\left(c_{x},c_{y}\right)$. The motion vector and velocity can be computed through the first-order difference as

(11) $$v_{t}=\left(c_{x}^{t}-c_{x}^{t-1},\;c_{y}^{t}-c_{y}^{t-1}\right),\;s_{t}=∥v_{t}{∥}_{2}$$

To minimize noise and achieve smooth motion representation, the centroid trajectory is subjected to Gaussian filtering on an optional basis. Other geometrical parameters such as the aspect ratio and convex hull area are introduced to account for variations in posture and spread of the body. All these parameters together capture both spatial and temporal patterns of motion, which allow discriminating structured motions.

#### 3.5.2. Gait Energy Image

**Figure 10:**
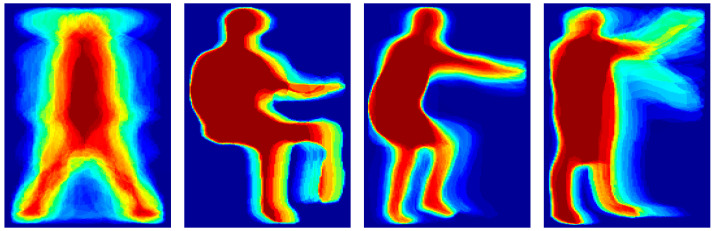
Gait Energy Images (GEIs) depicting spatio-temporal motion patterns, where high-intensity regions represent stable body positions and low-intensity regions indicate motion variability.

Gait Energy Image (GEI) has been used to characterize the spatio-temporal gait motion through silhouettes aggregated from multiple frames. Compared to other methods based on frames, GEI can represent both the static aspect as well as the dynamic aspect of human motion. Initially, binary silhouettes are extracted and spatially aligned using bounding-box normalization to ensure scale and position consistency across frames. Let $S_{t}(x,y)$ denote the aligned silhouette at time t. The GEI is computed as the mean intensity over N frames:

(12) $$\mathrm{GEI}\;\left(x,y\right)=\frac{1}{N}\sum_{t=1}^{N}S_{t}\left(x,y\right)$$

where the pixel intensities denote the number of times the body was present at that spatial location. Areas where the intensity values are higher are areas that are consistent with the body’s static body parts, whereas blurring denotes variation in motion. To improve clarity, contrast normalization through CLAHE is performed, after which the output is color mapped as shown in Figure 10. The generated GEI provides a comprehensive representation of the body’s position and movement over time. It makes it easy to distinguish between various activities because of its ability to preserve information despite temporal changes.

#### 3.5.3. Spatio-Temporal Graph Features (ST-GCN)

ST-GCN features utilize a spatio-temporal model for human motion by representing the skeleton as a graph composed of joints (nodes) and anatomical connections (edges). In this study, a reduced 17-joint configuration is adopted, and the spatial graph is decomposed into three subsets root, centripetal, and centrifugal based on directional relationships with respect to the body center.

(13) $$A_{norm}=D^{-1/2}\left(A+I\right)D^{-1/2}$$

**Figure 11:**
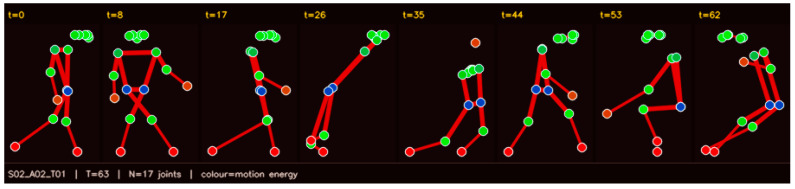
Temporal sequence of skeleton graphs illustrating joint connectivity and motion evolution across frames. Node colors represent motion energy, highlighting dynamic joints and activity intensity over time.

Given input $X\in R^{T\times N\times C}$, spatio-temporal graph convolution is applied as

(14) $$H_{t}=\sigma \left(A_{norm}X_{t}W\right)$$

where $X_{t}\in R^{N\times C}$ denotes the node-feature matrix at time step t, $A_{norm}\in R^{N\times N}$ is the normalized adjacency matrix, W represents the learnable graph-convolution weight matrix, and σ(⋅) denotes the ReLU activation function. Graph convolution is applied independently to each temporal frame, and the resulting frame-level embeddings are subsequently aggregated to form the spatio-temporal representation as illustrated in Figure 11.

#### 3.5.4. Spatial–Temporal Transformers (ST-TR)

Spatio-Temporal Transformer (ST-TR) features model skeleton sequences using self-attention to capture global joint and temporal dependencies. Given $X\in R^{T\times N\times C}$, joint coordinates and velocities are projected into a latent space $R^{D}$ with added spatial and temporal positional encodings. Spatial self-attention (SSA) operates across joints, while temporal self-attention (TSA) captures frame-wise dependencies.

(15) $$\mathrm{Attention}\left(Q,K,V\right)=\mathrm{softmax}\left(\frac{QK^{T}}{\sqrt{d_{k}}}\right)V$$

**Figure 12:**
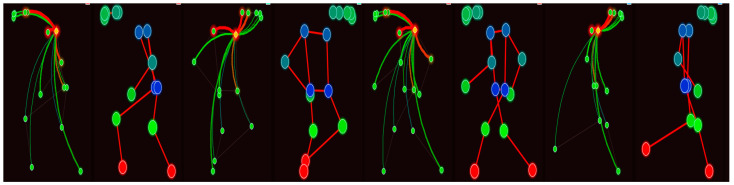
Spatial–temporal attention visualized on skeleton sequences, where arcs and node intensities indicate joint interactions and motion saliency across frames.

The resulting embeddings $Z\in R^{T\times N\times D}$ encode long-range interactions and are aggregated via global pooling and attention-based statistics. This representation effectively captures complex motion patterns and temporal dynamics for robust activity discrimination as shown in Figure 12.

Spatial–temporal attention visualized on skeleton sequences for a representative trial (S01_A01_T01, T = 60 frames), shown at six sampled time steps (t = 0, 11, 23, 35, 47, 59). For each time step, the left panel shows spatial self-attention (SSA)—arcs and node glow indicate joint-to-joint attention strength and the received attention, with the most-attended joint marked by a star—and the right panel shows temporal self-attention (TSA), where node size and color encode embedding magnitude and motion energy. Matching “t=” and color-tag labels confirm that the SSA/TSA pair in each column corresponds to the same frame. Although some sampled frames exhibit visually similar poses due to the smooth progression of the activity sequence, the displayed indices correspond to distinct temporal positions and no frame duplication or temporal offset is present.

### 3.6. Multimodal Feature Fusion

Finally, a multimodal fusion framework is applied that merges complementary visual and inertial features for a holistic description of actions. The aim is to leverage the spatial–structural properties of vision features and temporal–dynamic aspects of IMUs signals to achieve a higher discrimination capability in recognizing human activities.

Visual feature representations describe the motion characteristics of the actions using two modalities, that is, the full-body and skeleton-based motions. Orientation and trajectory features account for the posture and motion directions of the action execution. The Gait Energy Image (GEI) characterizes the spatio-temporal distributions of actions. Moreover, skeleton-based features using ST-GCN and ST-TR describe the relationships among joints, motions, and temporal dependencies in actions. These features collectively form a visual descriptor $F_{\upsilon }\;\epsilon \;R^{d_{\upsilon }}$, capturing posture, motion structure, and kinematic behavior.

Inertial feature representations can be extracted from the tri-axial accelerometer and gyroscope measurements. Handcrafted features TKEO, Fractal Dimension, and Permutation Entropy describe the dynamics and complexity in terms of energy. Frequency-domain and statistical descriptors provide information about the periodicity and distributions of signals. Moreover, 1D-CNN features model the hierarchical temporal patterns of actions. These components are aggregated into an inertial descriptor $F_{i}\;\epsilon \;R^{d_{i}}$, representing motion intensity, irregularity, and temporal evolution.

An early fusion scheme is then utilized by simply concatenating the two modality-specific feature vectors, thus yielding

(16) $$F_{joint}=\left[F_{v};F_{i}\right]\in R^{\left(d_{v}+d_{i}\right)}$$

In order to promote feature interactions and reduce redundancies, the concatenated feature vector is mapped to a lower-dimensional latent space with a fully connected layer using a nonlinear activation function.

(17) $$F_{fused}=\sigma \left(W_{f}F_{joint}+b_{f}\right)$$

where $W_{f}\in R^{d_{f}\times \left(d_{v}+d_{i}\right)}$, $b_{f}\in R^{d_{f}}$, and $\sigma \left(\cdot \right)$ denotes the ReLU activation. This transformation enables the model to learn cross-modal correlations and nonlinear interactions between visual and inertial features while reducing redundancy. The resulting fused representation $F_{fused}\in R^{d_{f}}$ serves as a compact and discriminative input for the subsequent attention-based classification stage, ensuring improved robustness across diverse activity patterns.

### 3.7. Ranger Optimizer

Following feature fusion, the optimization of the attention-based classifier is performed using the Ranger optimizer, which integrates Rectified Adam (RAdam) and Lookahead to ensure stable convergence in the high-dimensional multimodal feature space. The fused representation $F_{fused}\in R^{d_{f}}$ introduces complex correlations across modalities, resulting in a non-convex loss landscape where standard optimizers often exhibit unstable updates or slow convergence. Ranger addresses this by combining adaptive variance rectification with weight interpolation, improving both convergence speed and generalization.

The core update is governed by RAdam, which extends Adam by rectifying the variance of adaptive learning rates. Given gradients $g_{t}$, the first and second moment estimates are computed as

(18) $$m_{t}=\beta_{1}m_{t-1}+\left(1-\beta_{1}\right)g_{t},\;\;v_{t}=\beta_{2}v_{t-1}+\left(1-\beta_{2}\right)\;g_{t}^{2}$$

with $\beta_{1}=0.95$ and $\beta_{2}=0.999$. The variance rectification term is defined by using

(19) $$\rho_{t}=\rho_{max}-\frac{2t\beta_{2}^{t}}{1-\beta_{2}^{t}},\;r_{t}=\sqrt{\frac{\left(\rho_{t}-4\right)\left(\rho_{t}-2\right)\rho_{max}}{\left(\rho_{max}-4\right)\left(\rho_{max}-2\right)\rho_{t}}}$$

where $\rho_{max}=\frac{2}{1-\beta_{2}}-1$. When $\rho_{t}>5$, the parameter update is

(20) $$\theta_{t+1}=\theta_{t}-\eta \;r_{t}\frac{{\hat{m}}_{t}}{\sqrt{{\hat{v}}_{t}}+\epsilon }$$

Otherwise, a momentum update will be performed. This rectification stabilizes early training, particularly when learning cross-modal interactions in $F_{fused}$. Lookahead further improves optimization by maintaining fast weights ϕ and slow weights θ. After every k=6 inner update, slow weights are updated as

(21) θ←θ+α(ϕ−θ)

with interpolation factor α=0.5, followed by resetting ϕ←θ. This mechanism smooths optimization trajectories and avoids sharp minima, which are common when learning attention weights over heterogeneous features.

The optimizer is configured with a learning rate $\eta =10^{-3}$, weight decay $\lambda =10^{-2}$, and gradient clipping with maximum norm $||g|{|}_{2}\;\leq 1.0$ to suppress gradient spikes arising from attention layers. A batch size of 32 is used to balance gradient stability and computational efficiency. These choices ensure controlled updates of both projection weights $W_{f}$ and attention parameters, preventing overfitting and improving feature separability.

The combined effect of variance rectification and weight interpolation enables stable training under multimodal noise conditions, where inconsistencies in visual silhouettes and sensor signals may introduce irregular gradients. This optimization strategy ensures reliable convergence and robust learning of discriminative representations for activity classification.

Ranger was selected instead of conventional optimizers because the combination of Rectified Adam (RAdam) and Lookahead improves convergence stability and generalization when learning from high-dimensional multimodal feature representations.

**Figure 13:**
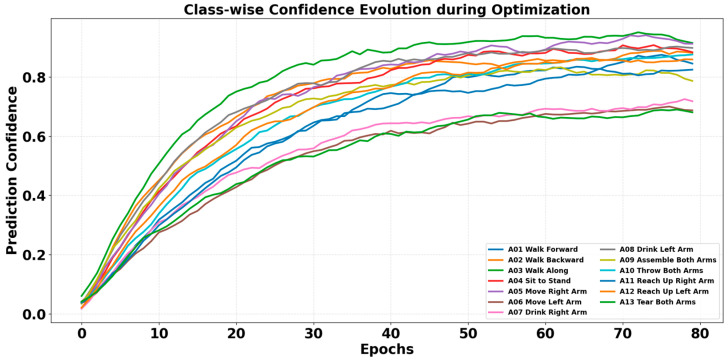
Ranger optimization results for VIDIMU dataset.

Figure 13: Class-wise confidence evolution during Ranger optimization on the VIDIMU dataset over 80 training epochs. Confidence values represent full-epoch average confidence scores for each of the 13 activity classes (A01–A13). Left- and right-arm variants of the movement activities (A05/A06, A07/A08, and A11/A12) are labeled separately to reflect the distinct activity definitions provided in the VIDIMU protocol.

### 3.8. Classification

**Figure 14:**
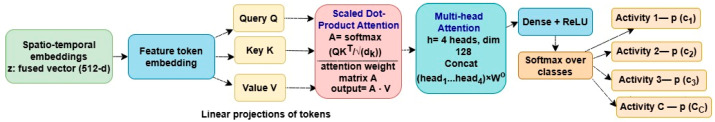
Architecture of attention-based classification network.

Following feature fusion and optimization, classification is performed using an attention-based neural architecture designed to selectively emphasize the most informative components of the fused representation as shown in Figure 14. Given the fused feature vector $F_{fused}\in R^{d_{f}}$ (with $d_{f}=512$), the objective is to learn adaptive weighting over feature dimensions to enhance discriminative patterns relevant to activity classes. The attention mechanism is implemented as a feature-token self-attention module. The fused feature vector $F_{fused}\in R^{d_{f}}$ is reshaped into a token sequence $T\in R^{L\times d_{t}}$, where $Ld_{t}=d_{f}$. In our implementation, $d_{f}=512$, L=8, and $d_{t}=64$. Query, key, and value matrices are then computed from T, allowing self-attention to capture interactions among feature tokens within the fused representation. Specifically, the input is first projected into query, key, and value spaces:

(22) $$Q=TW_{Q},\;K=TW_{k},\;V=TW_{v}$$

where $W_{q},W_{k},W_{v}\in R^{d_{f}\times d_{a}}$ are learnable projection matrices and $d_{a}=128$ denotes the attention embedding dimension. Consequently, $Q,K,V\in R^{L\times d_{a}}$. The attention weights are then computed using scaled dot-product attention:

(23) $$A=\mathrm{softmax}\left(\frac{QK^{T}}{\sqrt{d_{a}}}\right)\in R^{L\times L}$$

where A $\in R^{L\times L}$ represents the attention matrix that quantifies pairwise interactions among the feature tokens within the fused representation.

(24) $$F_{att}=AV{\in R}^{L\times d_{a}}$$

The resulting attention-enhanced representation $F_{att}$ captures contextual relationships among the tokenized sub-components of the fused multimodal embedding and enables adaptive emphasis of informative feature interactions. This mechanism allows the model to dynamically reweight feature-token contributions, emphasizing salient cross-modal interactions while suppressing redundant or noisy components introduced during fusion. To improve representation capacity, a multihead configuration with h = 4 heads is employed, enabling the model to attend to different subspaces of the token embeddings. The outputs of all heads are concatenated and linearly projected:

(25) $$F_{mh}=\mathrm{Concat}\left(F_{att}^{\left(1\right)},\ldots ,F_{att}^{\left(h\right)}\right)W_{o}$$

where $W_{o}$ is a learnable output projection matrix. The resulting representation is reshaped back to the fused feature space prior to the residual connection and layer normalization step. A residual connection and layer normalization are applied to stabilize training:

(26) $$F_{out}=\mathrm{LayerNorm}\left(F_{fused}+F_{mh}\right)$$

The resulting representation is passed through a feed-forward classification head consisting of two fully connected layers with dimensions 512→256→C, where C is the number of activity classes. ReLU activation is applied in the hidden layer, followed by Softmax for probability estimation:

(27) $$\hat{y}=\mathrm{softmax}\left(W_{2}\;\sigma \left(W_{1}F_{out}+b_{1}\right)+b_{2}\right)$$

To prevent overfitting, dropout with rate p=0.3 is applied after the attention and hidden layers. The model is trained using categorical cross-entropy loss:

(28) $$L=-\sum_{c=1}^{C}y_{c}log\left({\hat{y}}_{c}\right)$$

This attention-based formulation is particularly suited to the proposed multimodal framework, as it explicitly models interdependencies between visual and inertial features. By assigning adaptive attention weights across the tokenized multimodal representation, the classifier can focus on critical motion cues while reducing the influence of modality-specific noise. The integration with Ranger optimization further ensures stable convergence of attention parameters, leading to improved generalization across diverse activity patterns.

The parameters of attention-based classifier are represented in Table 1. The attention mechanism was incorporated to dynamically prioritize informative multimodal features and reduce the influence of redundant or noisy information originating from heterogeneous sensing modalities.

## 4. Performance Evaluation

The system in question was examined using two common benchmark datasets. Its performance was thoroughly investigated by confusion matrices, precision and recall metrics, F1 scores, and Receiver Operating Characteristic (ROC) curves, collectively highlighting its usefulness. The evaluation was conducted independently on the VIDIMU and UTD-MHAD datasets using five-fold cross-validation. The available samples were randomly partitioned into five approximately equal folds, with four folds used for training and one fold used for testing in each iteration. The final performance metrics were obtained by averaging the results across all folds. The framework was evaluated independently on each dataset; training was not performed on one dataset and tested on the other.

### 4.1. Dataset Description

#### 4.1.1. VIDIMU Dataset

The VIDIMU dataset [5] is an example of a multimodal kinematics dataset that was created for the purpose of recognizing human activities and analyzing their biomechanics, featuring synchronized videos and inertial measurements of 54 healthy young individuals; furthermore, out of them, 16 were equipped with wearable IMUs. The set contains 13 clinically important activities related to the movements of both the upper and lower limbs. The dataset features recordings of the movements, captured by a monocular RGB camera at approximately 30 Hz and recorded with the use of five body-worn IMUs at roughly 50 Hz. Video files feature 3D coordinates of joints, whereas IMU data is converted to joint angles using inverse kinematics. In contrast to lab-based datasets, this dataset is characterized by natural movement in home-like environments obtained with inexpensive sensors.

#### 4.1.2. UTD-MHAD Dataset

The UTD-MHAD dataset [12] contains 861 instances of data from eight individuals and has 27 classes of actions starting from gestures including wave and clap to bodily actions that include ArmCross, ArmCurl, BaseballSwing, BasketballShoot, Bowling, Boxing, Catch, Clap, CLW and CCLW (Draw Circle Clockwise and Draw Circle Counter Clockwise), DrawTriangle, DrawX, Jog, Knock, Lunge, PickUpandThrow, Push, SitToStand, Squat, StandToSit, SwipeLeft, SwipeRight, TennisServe, TennisSwing, Throw, Walk, and Wave. The dataset provides synchronized RGB-D and IMU recordings, providing the opportunity for multimodal action recognition in structured action categories and balancing the gender distribution. In our experiment, we used precision, recall, and F1 score for evaluation of metrics quantitatively. These metrics provide a clear measure of consistency of models with respect to class distributions and particularly help when dealing with an imbalanced or multiple class action dataset.

Although VIDIMU and UTD-MHAD were collected under controlled experimental settings and primarily involved healthy participants, they contain diverse movement patterns, biomechanical actions, and synchronized multimodal sensor streams that are directly relevant to athlete-performance monitoring and rehabilitation assessment. Nevertheless, they may not fully capture the variability observed in real-world sports and clinical environments, including differences in training intensity, fatigue-related movement adaptations, injury status, rehabilitation progression, sensor-placement variations, and environmental conditions. Consequently, these datasets provide suitable benchmark platforms for evaluating the proposed framework, while future validation on athlete-specific and real-world rehabilitation cohorts will further strengthen its practical applicability and generalizability.

## 5. Results and Analysis

In this section, several experiments have been carried out for the assessment of the suggested system. For the assessment, the following measures were considered: confusion matrix, precision, recall, F1 measure, and ROC curve. The detailed results of these experiments are discussed below.

### 5.1. Experiment 1: Confusion Matrix

**Figure 15:**
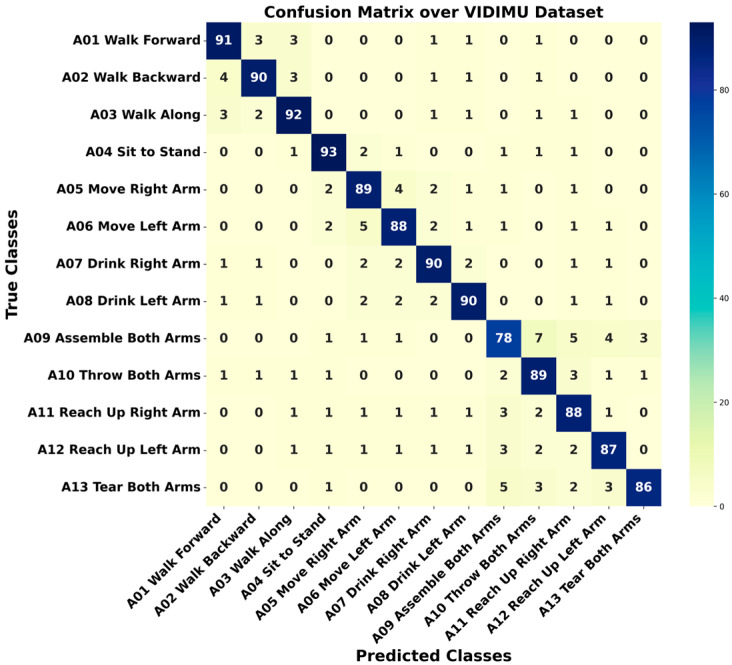
Confusion matrix for various classes for VIDIMU dataset.

**Figure 16:**
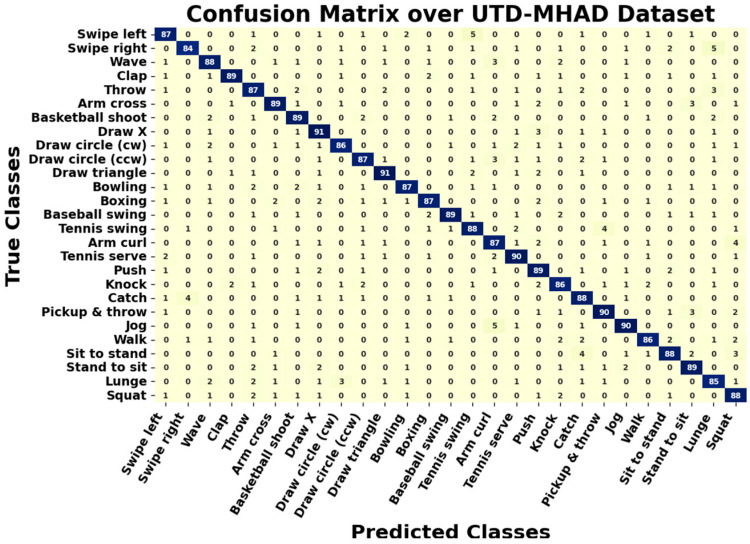
Confusion matrix for various classes for UTD-MHAD dataset.

In the first experiment, we plotted the confusion matrix for both datasets. The confusion matrix gives a concise visual representation of the classifier’s performance, emphasizing its strengths and limitations in terms of how it handles different classes. Figure 15 and Figure 16 exhibit the confusion matrix for the VIDIMU and UTD-MHAD datasets, respectively.

### 5.2. Experiment 2: Precision, Recall and F1 Score

**Figure 17:**
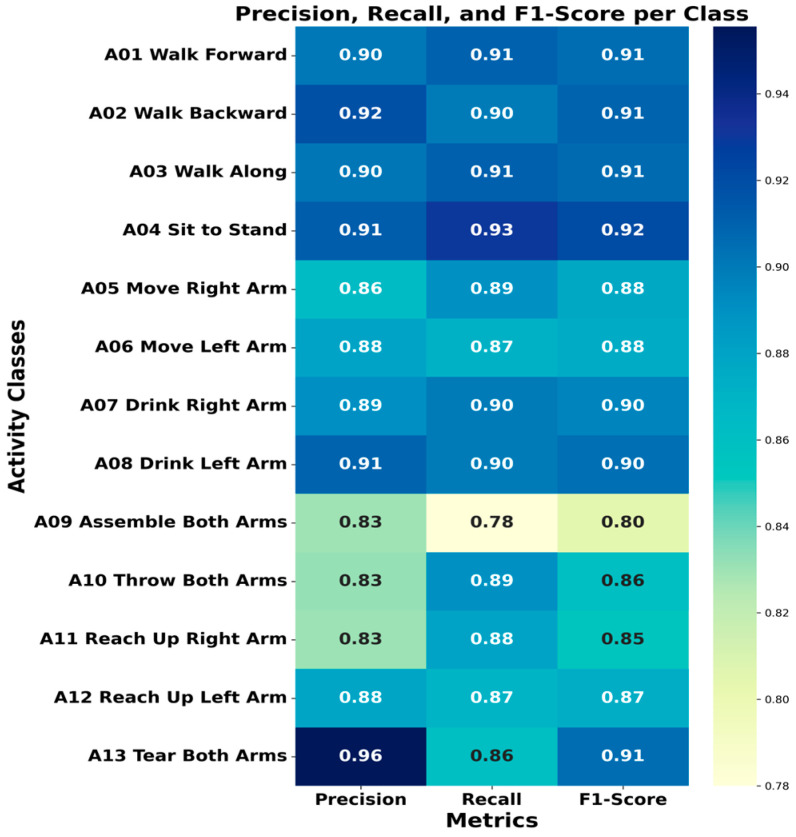
Evaluation matrices (precision, recall and F1 score) for various classes for VIDIMU dataset.

**Figure 18:**
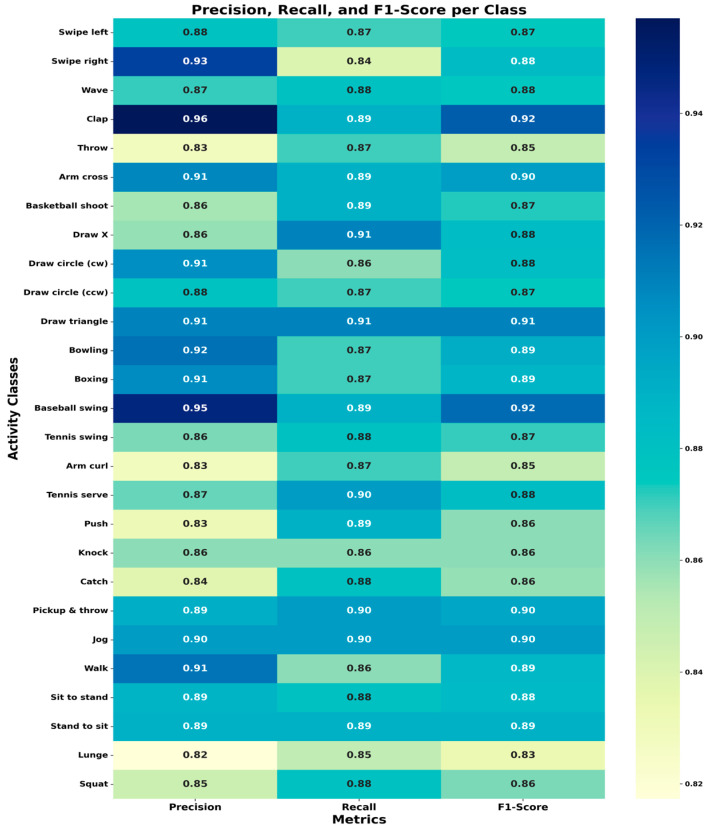
Evaluation matrices (precision, recall and F1 score) for various classes for UTD-MHAD dataset.

In this experiment, the proposed system undergoes a thorough evaluation, accompanied by an in-depth analysis of its specific implications in certain domains. Figure 17 and Figure 18 present the evaluation matrices, including precision, recall and F1 score for VIDIMU and UTD-MHAD datasets, respectively.

Despite the promising results, several limitations should be considered. Recognition performance may be affected when activities exhibit highly similar movement patterns or when sensor measurements are degraded by occlusions, motion artifacts, or variations in sensor placement. Furthermore, although multimodal fusion improves robustness, real-world deployments may introduce additional variability not fully represented in benchmark datasets. These factors motivate future investigations into adaptive domain-generalization and personalized activity-recognition strategies.

## 6. Discussion and Analysis

Evaluation results of the proposed multimodal framework for athlete-performance analysis and rehabilitation monitoring on the UTD-MHAD and VIDIMU datasets demonstrate consistently strong performance across diverse activity categories. For the VIDIMU dataset, locomotion and transitional activities such as “Walk Forward,” “Walk Backward,” “Walk Along,” and “Sit to Stand” achieve well-balanced precision and recall values, resulting in F1 scores of approximately 0.91–0.92. Similarly, upper-limb activities including “Move Right Arm,” “Move Left Arm,” “Drink Right Arm,” and “Drink Left Arm” are recognized with F1 scores ranging from 0.88 to 0.90, indicating effective modeling of fine-grained motion patterns and coordinated arm movements. The comparatively lower performance observed for “Assemble Both Arms” (F1 ≈ 0.80) can be attributed to the subtle and low-amplitude motion characteristics of the activity, which increase its similarity to other upper-limb actions. “Throw Both Arms” and the reach-up activities also exhibit slightly lower recognition rates (F1 ≈ 0.85–0.86), likely due to higher intra-class variability and overlapping motion trajectories. Nevertheless, all VIDIMU activities achieve F1 scores above 0.80, demonstrating robust recognition performance across both locomotor and manipulation-oriented tasks.

For the UTD-MHAD dataset, the proposed framework achieves similarly strong performance, with most activities attaining F1 scores between 0.85 and 0.92. Distinctive activities such as “Clap” (F1 ≈ 0.92), “Baseball Swing” (F1 ≈ 0.92), and “Draw Triangle” (F1 ≈ 0.91) are recognized with high accuracy owing to their characteristic motion patterns. Activities such as “Jog,” “Pickup & Throw,” and “Tennis Serve” maintain F1 scores close to 0.90, indicating stable recognition of both cyclic and complex whole-body movements. Slightly lower performance is observed for activities including “Lunge” (F1 ≈ 0.83), “Throw” (F1 ≈ 0.85), and “Arm Curl” (F1 ≈ 0.85), which may be explained by greater intra-class variability and biomechanical similarities with other actions. Analysis of the confusion matrices further indicates that most misclassifications occur between activities exhibiting similar movement semantics or kinematic characteristics, suggesting that the model learns meaningful activity representations rather than overfitting dataset-specific patterns.

Aside from being important with regard to the efficiency of recognizing activities, the presented system may also have some interesting applications for monitoring athletes as well as analyzing movement patterns related to rehabilitation purposes. Indeed, accurate activity recognition enables a way to detect any abnormalities in movement, indicating poor efficiency, compensation due to fatigue, or a greater possibility of injury. In sport-related situations, constant tracking of how activities are performed as well as transitions between them may help evaluate the workload or train athletes. The same way, for patients who are undergoing rehabilitation treatment, consistent recognition of their movements and activities can aid clinicians in evaluating their compliance, progress, and general movement pattern quality. While currently focused on recognizing activities rather than using that information for other tasks, the multimodal representations used in the proposed framework may become important for assessing risks of injuries, detecting fatigue, tracking rehabilitation progress, and evaluating movement patterns themselves. The integration of wearable sensing and vision-based analysis provides a practical foundation for intelligent sports analytics, enabling continuous monitoring of movement quality, workload patterns, training execution, and biomechanical efficiency. Such capabilities are increasingly important in modern sports environments that rely on objective performance metrics to support coaching decisions, injury prevention, and rehabilitation management.

Performance analysis confirms the results obtained previously in other HAR research, where actions associated with unique movement features have usually been recognized more accurately compared to those that involve subtle or overlapped movement features. It seems that the methodology described works effectively for modeling global movement structure as well as movement dynamics on a smaller scale due to the use of multimodal representation. This multimodal representation could probably explain the good balance between accuracy and recall for most classes.

### 6.1. Experiment 3: ROC (Receiver Operating Characteristic Curve)

**Figure 19:**
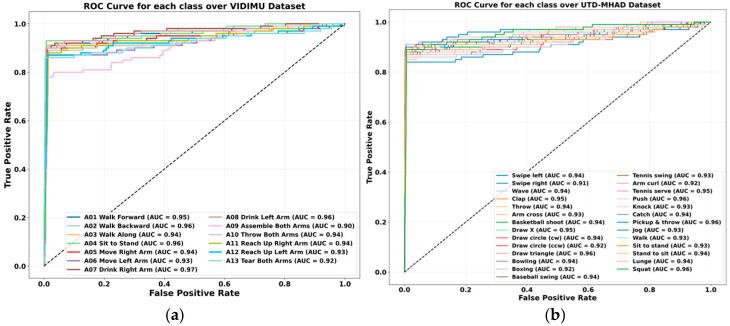
ROC curves for (**a**) VIDIMU and (**b**) UTD-MHAD datasets.

The ROC curves shown in Figure 19a,b describe the behavior of a categorization algorithm for health-related activities. The area under the curve (AUC) can be used as a performance indicator for the whole process, where an AUC closer to 1 show that the classifier is able to distinguish the target positive class (activity) from other activities.

### 6.2. Discussion and Analysis of ROC Curve

The Receiver Operating Characteristic (ROC) curves shown in Figure 19a and Figure 19b illustrate the class-wise discriminative capability of the proposed multimodal framework for athlete-performance analysis and rehabilitation monitoring on the VIDIMU and UTD-MHAD datasets, respectively. For the VIDIMU dataset, most activities achieve Area Under the Curve (AUC) values ranging from approximately 0.90 to 0.97, indicating strong class separability across different decision thresholds. Activities such as “Drink Right Arm” and “Drink Left Arm” achieve some of the highest AUC values (≈0.97 and ≈0.96, respectively), reflecting the consistent motion patterns associated with these actions. Locomotion and transitional activities, including “Walk Forward,” “Walk Backward,” “Walk Along,” and “Sit to Stand,” also demonstrate strong discriminative performance with AUC values between approximately 0.94 and 0.96. Comparatively lower AUC values are observed for “Assemble Both Arms” (≈0.90), “Move Left Arm” (≈0.93), and “Reach Up Left Arm” (≈0.93), which may be attributed to subtle motion characteristics and greater similarity to other upper-limb activities. Nevertheless, all VIDIMU activities achieve AUC values above 0.90, demonstrating robust recognition capability across diverse movement categories.

For the UTD-MHAD dataset, a similarly consistent pattern is observed, with most activities achieving AUC values between approximately 0.91 and 0.96. Activities characterized by highly distinctive motion patterns, such as “Clap,” “Draw Circle (CCW),” and “Squat,” achieve some of the highest AUC values (≈0.95–0.96). “Baseball Swing” also demonstrates strong discriminative performance with an AUC of approximately 0.94, indicating effective recognition despite exhibiting slightly lower-class separability than the highest-performing activities. Slightly lower AUC values are observed for activities exhibiting greater execution variability or kinematic similarity to other actions, including “Swipe Right,” “Arm Curl,” and “Tennis Serve” (≈0.91–0.92). Importantly, all ROC curves remain substantially above the diagonal reference line corresponding to random classification, confirming strong discriminative performance across all activity classes.

Overall, the ROC analysis demonstrates that the proposed framework achieves consistently high-class separability on both datasets, with only minor performance variations observed for activities sharing similar semantic characteristics or biomechanical movement patterns.

### 6.3. Experiment 4: Comparisons with Baseline Classifiers

A comparative analysis of the proposed approach relative to the baseline classifiers is summarized in Table 2 below. It can be observed from the results that conventional machine learning techniques like SVM and AdaBoost have relatively poor performance because of their inability to learn intricate spatio-temporal relations. Ensemble techniques like Random Forest show better performance than the aforementioned conventional classifiers but still fall behind the proposed approach. On the other hand, the CNN classifier attains better performance than the mentioned classifiers owing to its ability to learn hierarchical feature representations; however, its performance is relatively poor compared to the proposed approach.

Statistical reliability was assessed using five-fold cross-validation. The reported values represent the mean classification accuracy ± standard deviation together with the corresponding 95% confidence intervals. The reported confidence intervals indicate consistent performance improvements of the proposed framework across repeated experimental runs.

The effectiveness of the proposed framework is largely due to the complementary nature of the individual modules that make up the framework. The preprocessing steps enhance the quality and consistency of signals, the hybrid feature extraction algorithm extracts a wide range of biomechanical and spatio-temporal features, multimodal fusion utilizes the complementary strengths of the different sensor modes, the Ranger optimization increases the stability of learning processes, and the attention-based classification increases discrimination through the selection of important features.

### 6.4. Experiment 5: Comparisons with Existing Studies

Table 3 shows the comparison with existing studies. Some critical points raised through the comparison of results given in Table 3 include: early models built on the principles of individual sensing techniques were proven to produce accurate results in their specific applications but lacked some key aspects in their design. For example, IMU systems [8] provided sufficient information about movements but did not account for visual context, whereas EMG solutions [10,11] offered detailed information about neuromuscular processes in the body but required proper electrode placements and were sensitive to inter-subject variations. In addition, vision-based methods [19] allowed non-contact sensing but were susceptible to occlusions, illumination variations, and viewpoint changes.

Most of the existing multimodal solutions [13,14,15,16,17,18] show that a combination of various sensing modalities typically leads to higher robustness and classification quality. Nevertheless, most current multimodal studies are concerned mainly with fusion and classification and pay much less attention to the development of algorithms for modality-specific data preprocessing, adaptive segmentation, hybrid features extraction, and attentive feature selection. Moreover, most existing frameworks use only machine-learned features and ignore the benefits that could be gained from handcrafted biomechanical descriptors accounting for different properties of movements like energy, complexity, or regularity.

There are several factors contributing to enhanced accuracy rates obtained using the proposed framework: first, the employment of RGB and inertial sensing allows complementary spatial and temporal modeling of postures and movements, respectively. Second, the combination of machine-learned representations with handcrafted features such as Teager–Kaiser Energy Operator, Fractal Dimension, and Permutation Entropy helps the network better understand the biomechanics of movements. Third, adaptive segmentation and modality-specific preprocessing help increase signal consistency prior to feature extraction. Fourth, attention mechanisms and Ranger optimizers help select the best features for classification more effectively.

However, there are still some limitations. Firstly, activities involving subtle movements, a high degree of intra-class variation, and similar motion trajectories might still prove difficult to classify. Secondly, even though the proposed system reached 88.40% and 87.96% accuracy rates on the VIDIMU and UTD-MHAD datasets, respectively, both databases were obtained under controlled conditions and cannot fully reflect real-world rehabilitation and sport environments. Various factors including displacements in sensors positions, athletes’ fatigue, injury-induced movement adjustments, environmental variability, and biomechanical diversity might affect classification in practice.

The obtained results are aligned with scientific knowledge regarding multimodal movement analysis, athlete monitoring, and rehabilitation assessment. In particular, it is known that unimodal recognition approaches usually underperform compared to the combination of multiple sensing modalities due to the complementarity of their properties. While vision-based methods have proven to be good sources of spatial data, they are highly dependent on changes in lighting conditions, presence of occlusions, or viewing angle. Meanwhile, sensor systems are able to reliably detect temporal changes but do not include contextual information provided by camera footage. Multimodal solutions thus allow us to reach high levels of performance thanks to the combined power of each method. As it is demonstrated in Table 4, state-of-the-art approaches achieve accuracies between 80% and 87.5% in different sports and motion recognition tasks. The proposed model has yielded 88.40% on VIDIMU and 87.96% on UTD-MHAD, showing comparable or even better results than previous works. It can be explained by such key design elements as separate data preprocessing, automatic adaptation of segment lengths, fusion of handcrafted biomechanics with deep representations, and attention-based fusion. Finally, similarly to previous results, it has been found that activities featuring less pronounced movements or with large variability within classes are harder to recognize compared to others.

The present findings are consistent with previous studies reporting the benefits of multimodal sensing for movement analysis, athlete monitoring, and rehabilitation assessment. Ordóñez and Roggen [13] demonstrated that deep-learning architectures combining convolutional and recurrent layers can effectively fuse heterogeneous inertial sensor streams and achieve superior recognition performance compared with conventional approaches, providing a methodological foundation that was subsequently extended to genuinely multimodal systems integrating inertial and visual sensing. Similarly, Gu et al. [16] reported improved robustness and predictive capability using multimodal wearable sensing systems. The results obtained in this study further support these observations, as the integration of inertial and vision-based modalities achieved consistent recognition performance across both benchmark datasets. In contrast to many existing approaches that rely predominantly on deep-learned representations, the proposed framework additionally incorporates handcrafted biomechanical descriptors (TKEO, FD, and PE), enabling the model to capture complementary motion characteristics related to energy fluctuations, movement complexity, and signal irregularity. The observed class-wise performance and ROC indicate that this hybrid representation strategy contributes to robust discrimination across diverse activity categories.

### 6.5. Experiment 6: Ablation Study

To investigate the contribution of individual components within the proposed framework, an ablation study was conducted by progressively removing or adding key modules, including visual features, inertial features, multimodal fusion, Ranger optimization, and the attention mechanism, as shown in Table 5.

The ablation results confirm that each component of the proposed framework contributes to the final recognition performance. The most significant performance reductions were observed when either the RGB modality or the IMU modality was removed, resulting in accuracy drops of 5.40% and 6.80% on VIDIMU and 4.80% and 5.60% on UTD-MHAD, respectively. These findings demonstrate that visual and inertial modalities provide complementary information and validate the effectiveness of multimodal fusion. Among the remaining components, the removal of handcrafted inertial descriptors produced the largest performance degradation, indicating that TKEO, Fractal Dimension, and Permutation Entropy provide valuable biomechanical information beyond deep-learned representations. Similarly, replacing Ranger with Adam, removing VMD preprocessing or adaptive windowing, and eliminating the attention mechanism all resulted in measurable performance reductions, confirming the contribution of optimization, preprocessing, and attention-guided feature selection to the overall framework. Collectively, these results demonstrate that the final performance is achieved through the synergistic integration of all proposed components rather than reliance on any single module.

## 7. Conclusions

In conclusion, this study highlights that athlete-performance analysis and rehabilitation monitoring extends beyond a conventional classification task and should instead be viewed as an integrated activity recognition framework combining wearable sensing, computer vision, and artificial intelligence for performance assessment. The proposed multimodal framework effectively fused information from inertial sensors and RGB images through modality-specific preprocessing, hybrid feature learning, feature fusion, and attention-driven deep learning techniques to capture complex spatio-temporal movement characteristics. By integrating wearable sensing and visual motion analysis, the framework provides a robust foundation for intelligent movement-monitoring systems capable of supporting biomechanical assessment, rehabilitation evaluation, and sports-performance assessment. Experimental results demonstrated that the proposed system achieved classification accuracies of 88.40% and 87.96% on the VIDIMU and UTD-MHAD datasets, respectively, outperforming the baseline machine-learning and deep learning models evaluated in this study, including Random Forest (RF), Support Vector Machine (SVM), Convolutional Neural Network (CNN), and AdaBoost architectures. Furthermore, the incorporation of silhouette-refined visual descriptors, skeleton representations, segmented inertial signals, and Ranger-based optimization improved feature separability, model convergence, and recognition robustness under varying movement execution and sensing conditions. The attention-driven architecture further demonstrated superior capability in extracting informative multimodal representations, highlighting the importance of attention-based learning for movement-focused artificial intelligence systems in sports and rehabilitation environments. Beyond activity recognition, the proposed framework offers a foundation for intelligent sports-monitoring systems by enabling continuous assessment of movement quality, biomechanical efficiency, training execution, and rehabilitation progression through multimodal sensing.

While the performance of the suggested framework was impressive on the VIDIMU and UTD-MHAD benchmarking datasets, some limitations related to the potential generalizability to the real world should be mentioned. VIDIMU presents multimodal data collected during movements under more natural conditions, which are relevant to the needs of the motion analysis within the scope of rehabilitation. At the same time, UTD-MHAD can be considered as a standardized multimodal benchmark for the assessment of the performance in recognizing different types of movements. Hence, they do not necessarily reflect the variability of sports and rehabilitation contexts in terms of injuries, progress, movement issues, fatigue, sensor placements, and environmental factors.

Considering the applications of the framework, it is better to see the proposed framework as an enabler technology for intelligent systems that monitor athletes’ movements and aid rehabilitation rather than just being used for activity recognition. In combining wearable sensors, computer vision, and attention learning, the framework provides the system with the capability to monitor athletes continuously, which could be utilized to assess movement quality, record rehabilitation exercises, evaluate training performance, and detect movements that are indicative of fatigued states or potential injury risks. Future research work will extend the framework in clinical and sport-related outcome measures through longitudinal and personal movement assessment.

Overall, the key contribution of this work lies in demonstrating how multimodal sensing, computer vision, and deep learning can be integrated within a unified framework to support next-generation digital platforms for movement assessment, rehabilitation tracking, and sports-performance evaluation. The interaction between wearable technologies, visual motion tracking, and AI-driven analytics reflects the growing transition toward data-driven and athlete-centered approaches in modern sports science and rehabilitation applications. Within this context, intelligent multimodal HAR systems can enable continuous activity recognition, athlete-movement assessment, rehabilitation support, injury-risk mitigation, and performance optimization across sports and healthcare environments. Such technologies also support the broader evolution of digital sports and healthcare platforms, where real-time movement analytics and intelligent sensing frameworks are increasingly used to improve athlete development, rehabilitation outcomes, and long-term physical performance management. Future work should therefore focus on real-time implementation, scalable multimodal learning architectures, and generalized cross-domain frameworks capable of addressing the challenges associated with real-world sports and rehabilitation applications.

## Figures and Tables

**Table 1:** Attention-Based Classification Parameters over VIDIMU dataset.

Parameters Name	Values
Initial Learning Rate	$1\times 10^{-3}$ (adaptive via Ranger)
Epochs	50
Batch Size	32
Dataset Split	5-fold cross-validation
Activation Function	ReLu (hidden layers), Softmax (output layer)
Optimizer	Ranger
Attention Heads	4
Attention Dimension	128
Fused Feature Dimension	512
Dropout Rate	0.3
Weight Decay	$1\times 10^{-2}$

**Table 2:** Comparison of our model with baseline classifiers under the same experimental setup for VIDIMU and UTD-MHAD datasets.

	Method		Accuracy %	
	VIDIMU	UTD-MHAD	95% CI (VIDIMU)	95% CI (UTD-MHAD)
SVM	74.50 ± 2.21	76.20 ± 2.43	[71.75, 77.25]	[73.17, 79.22]
Random Forest	78.10 ± 2.17	79.30 ± 2.51	[75.41, 80.79]	[76.17, 82.42]
CNN	84.20 ± 2.30	85.10 ± 2.26	[81.34, 87.06]	[82.30, 87.90]
AdaBoost	72.80 ± 0.99	74.60 ± 2.41	[71.57, 74.02]	[71.61, 77.58]
**Proposed**	**88.40** ± **1.44**	**87.96** ± **2.07**	**[86.61, 90.19]**	**[85.40, 90.53]**

Bold values indicate the best performance and denote the proposed framework.

**Table 3:** Comparison of the proposed framework with representative multimodal studies.

Ref.	Sensor Modality	Method	Main Contribution	Key Limitations
[9]	ECG	Deep neural network for arrhythmia detection	Automated cardiovascular monitoring	Limited to ECG signals; does not capture movement or biomechanical information
[8]	IMU	Wearable inertial sensing for lower-limb rehabilitation	Joint angle and gait assessment	Restricted physiological context; depends on wearable sensor quality and placement
[10]	EMG	EMG-driven rehabilitation monitoring	Muscle activity assessment and biofeedback	Sensitive to electrode placement and signal noise
[11]	EMG	Neurorehabilitation monitoring	EMG-based rehabilitation support	Poor robustness across subjects and sessions
[19]	RGB camera	Pose estimation for remote physiotherapy	Vision-based rehabilitation assessment	Sensitive to illumination changes, occlusions, and camera viewpoints
[13]	Multimodal wearable sensors	CNN-LSTM multimodal HAR	Deep multimodal activity recognition	Focused mainly on deep fusion without modality-specific preprocessing
[16]	Multimodal wearables	Machine learning-based risk prediction	Fall-risk and mobility monitoring	Limited focus on detailed activity recognition and movement biomechanics
[15]	EMG + ECG + Motion Sensors	Bio-signal–motion integration	Comprehensive rehabilitation assessment	Primarily rehabilitation-oriented; limited activity recognition capabilities
[14]	Multimodal Rehabilitation Sensors	Intelligent rehabilitation systems	Adaptive rehabilitation feedback	Limited integration of advanced activity-specific feature learning
[17]	Wearables + EEG	Multisensor fusion with deep learning	Health monitoring and prediction	Focused on health feedback rather than fine-grained HAR
[18]	ECG + Heart Rate + Respiration + Alcohol Sensors	Emergency monitoring framework	Real-time health emergency detection	Not designed for human activity recognition or biomechanical analysis
Proposed	RGB + IMU	Hybrid handcrafted and deep features, multimodal fusion, Ranger optimization, attention-based classification	Athlete-performance analysis and rehabilitation-monitoring framework	Computational complexity is higher than unimodal systems; evaluation limited to benchmark datasets and controlled environments

**Table 4:** Comparison with existing studies.

Paper	Accuracy (%)
[20]	80.2
[21]	84.3–87.5
[22]	85.2
[23]	56.3–70
[21]	42.5–42.7

**Table 5:** Ablation study comparing unimodal and multimodal configurations of the proposed framework on the VIDIMU and UTD-MHAD datasets.

Configuration	VIDIMU Drop	UTD-MHAD Drop
w/o VMD Preprocessing	−2.80%	−2.60%
w/o adaptive windowing	−2.20%	−1.90%
w/o Handcrafted features (TKEO + FD + PE)	−3.10	−2.90
w/o Ranger → Adam	−1.80	−1.60
w/o Attention → FC	−2.50	−2.30
w/o RGB (IMU only)	−5.40	−4.80
w/o IMU (RGB only)	−6.80	−5.60

## Data Availability

The data presented in this study are available in VIDIMU at https://www.emergentmind.com/topics/vidimu-dataset (Access Date: 13 January 2026). These data were derived from the following resources available in the public domain: https://www.emergentmind.com/topics/vidimu-dataset (Access Date: 2 January 2026).; The data presented in this study are available in UDT-MHAD at https://personal.utdallas.edu/~kehtar/UTD-MHAD.html dataset (Access Date: 26 December 2025). These data were derived from the following resources available in the public domain: https://personal.utdallas.edu/~kehtar/UTD-MHAD.html (Access Date: 16 January 2026).
